# Fruit Quality Monitoring with Smart Packaging

**DOI:** 10.3390/s21041509

**Published:** 2021-02-22

**Authors:** Arif U. Alam, Pranali Rathi, Heba Beshai, Gursimran K. Sarabha, M. Jamal Deen

**Affiliations:** Department of Electrical and Computer Engineering, McMaster University, Hamilton, ON L8S 4L8, Canada; alamau@mcmaster.ca (A.U.A.); rathipg@mcmaster.ca (P.R.); beshai.heba08@gmail.com (H.B.); sarabhag@mcmaster.ca (G.K.S.)

**Keywords:** smart packaging, intelligent packaging, active packaging, fresh produce, fruit quality, fruit processing, fruit smart packaging, fruit sensor, RFID, IoT

## Abstract

Smart packaging of fresh produce is an emerging technology toward reduction of waste and preservation of consumer health and safety. Smart packaging systems also help to prolong the shelf life of perishable foods during transport and mass storage, which are difficult to regulate otherwise. The use of these ever-progressing technologies in the packaging of fruits has the potential to result in many positive consequences, including improved fruit quality, reduced waste, and associated improved public health. In this review, we examine the role of smart packaging in fruit packaging, current-state-of-the-art, challenges, and prospects. First, we discuss the motivation behind fruit quality monitoring and maintenance, followed by the background on the development process of fruits, factors used in determining fruit quality, and the classification of smart packaging technologies. Then, we discuss conventional freshness sensors for packaged fruits including direct and indirect freshness indicators. After that, we provide examples of possible smart packaging systems and sensors that can be used in monitoring fruits quality, followed by several strategies to mitigate premature fruit decay, and active packaging technologies. Finally, we discuss the prospects of smart packaging application for fruit quality monitoring along with the associated challenges and prospects.

## 1. Introduction

Present civilization has an extreme urgency of innovation in the field of food production, storage, and distribution to solve hunger problems that still afflict several parts of the world. Food production is rapidly growing with the help of science and technology due to increased demand. However, there is a large disparity between the different countries where there are constant food shortages and some other countries where food is wasted at an alarming rate [1]. Despite the differences, all societies have a common need for better methods of preventing unnecessary food spoilage. Also, the global food market has a higher than ever demand to deliver fresher, higher quality food at a reasonable cost [2]. With a global population of 7.6 billion producing over 1.3 billion tonnes of food wastage, and a projected population of 9.6 billion by 2050 [3], current food processing practices and technologies must be assessed and improved.

Fruits and vegetables are living biological bodies, having a respiratory system that continues during their living processes and even after harvest. By respiration, fruits and vegetables take in oxygen and give out carbon dioxide. Therefore, the handling and packaging of fruits are key toward maintaining their freshness until they reach the consumer’s table. One of the major sources of premature spoilage in perishable fruits is during transport and storage. This is a key issue for fresh fruits, which gets very little quality monitoring after it has been harvested, treated, and packed. Fruits are necessary parts of the human diet, as they contain vitamins, minerals, antioxidants, fiber, and many other essential nutrients [4]. Most countries suggest daily servings of fruits and vegetables in their dietary recommendation guides, emphasizing the importance of accessibility to good quality produce [5]. The health and safety of the consumers are also important factors in the consideration of fruits quality. Many harmful microorganisms can infect fresh produce, especially through poor handling and processing practices. A majority of these pathogens are picked up during transport and storage and due to faulty packaging techniques [6]. Due to the potential severity of these hazards, regulations on fresh fruits are stringent, particularly in the developed countries. Resellers take precautionary measures such as disposing of food that has passed its “best before” date, which the United States Department of Agriculture states is not a true indicator of spoiled food [7]. Products are often still viable after this date, meaning that large amounts of unsold food are unnecessarily discarded [8].

Conventional packaging aims to limit the harmful environmental exposure of fruits and fruit products, but it is often not enough. It is difficult to monitor and retain the peak level of quality during all stages of food processing. In most cases, the fruit may not have warning about problems before it is too late. To address this need in produce processing, along with other food-processing innovations, freshness sensors and smart packaging system for fruit monitoring have emerged. Fruit freshness sensors or indicators can sense and inform the status of a fruit’s quality, such as freshness, ripeness, leak, microbial pathogens, and emitted gases, correlated to the safety of the food being consumed. Therefore, the freshness sensors can be defined as the on-package sensors or indicators that can sense the freshness of the food associated to the environment inside or outside of the package and advise about the quality and safety of the food [9]. On the other hand, smart packaging systems offer methods for both passive and active packaging solutions. Embedded with sensor technology that can detect changes in fruits’ health and environmental conditions that impact quality, intelligent packaging allows for the use of real-time monitoring until the product is delivered to the customer. In fact, smart packaging systems can be further applied to create response systems that can mitigate spoilage conditions and prolong the shelf-life of perishable items. While still in its early stages, the numerous opportunities for the applications of smart packaging technology in spoilage prevention for fruits is a key motivator for further research and development [2].

There have been many studies on the use of freshness sensors and smart packaging systems in food and beverage industries [6], as well as their applications in water quality monitoring [10,11,12]. A few studies also focused on freshness monitoring and controlled packaging of meat and animal products [4,9,13]. However, there has been very limited studies that focused on the freshness monitoring and sensing of fruits and fruit products. In fact, many of these studies focused on the freshness of produces [14,15,16,17], with less attention to fruits and fruit products. The high spoilage rate of fruits and the increased instances of diseases outbreaks related to fruits demands a comprehensive study on the state-of-the-art technologies and future research trends on freshness monitoring and smart packaging of fruits inside a package. Freshness sensors and smart packaging systems for fruit monitoring can also have great potential in terms of the presence of emerging contaminants such as plastic micropollutants migrating from the plastic packages [18]. Also, a comprehensive review on the freshness sensors and smart systems technologies for fruits monitoring will illustrate current challenges and outlook for the technology roadmaps towards the possibility of further commercial use of smart packaging in the fruits packaging industry.

In this review, we provide state-of-the-art discussion of freshness sensors as smart packaging technologies for fruit quality monitoring. In Section 2, we provided a short background on fruit classification, processing stages, and harvesting procedures to understand the important biological parameters that can affect fruit deterioration. In Section 3, we discuss smart packaging systems, their compatibility, challenges toward integration with freshness sensors, and their classification into active and intelligent packaging in term of sensing mechanisms, scavenging, and data processing along with several commercial state-of-art examples of each type. In Section 4, we discuss the challenges and prospects of fruits monitoring and associated opportunities, especially overcoming the challenges in bridging between freshness sensors and smart packaging systems. Finally, a conclusion is provided highlighting the importance of freshness monitoring and smart packaging systems in assessing freshness of fruits and fruit products toward reduced wastes and improved consumer health.

## 2. Freshness of Fruits Related to Classification, Stages, and Harvesting

**Figure 1:**
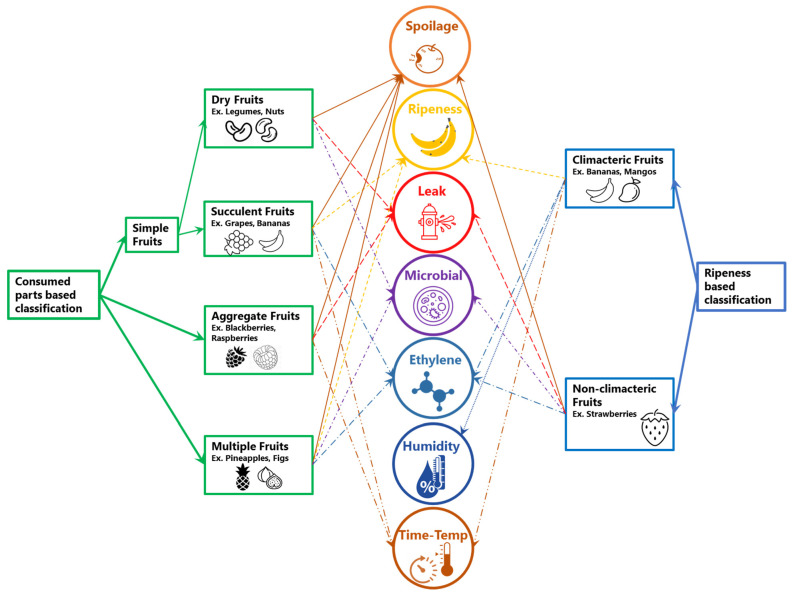
Fruit classification hierarchy and its relevance to different freshness parameters [19,20].

Freshness of fruits are governed by several aspects such as fruits classification, stages and harvesting procedure. Fruits are naturally occurring plant structures that provide nourishment for many animals, including humans. In this section, the classification and general development of fruit is discussed to provide a basis for the remainder of this review. When classifying plant anatomy, fruits are defined as the reproductive components. Produced from tissues of the plant’s flower structure, the fruit contains the seeds that will produce the next generation of botanical offspring. The process of pollination, where pollen is used to fertilize a plant’s ovum, marks the beginning of seed development. The surrounding ovary tissue, known as the pericarp, starts to develop into a protective layer that will be used to house the seeds. In most cases, the pericarp is the edible portion of the fruit. The fruit itself is classified into one of three categories: simple fruits, aggregate fruits, and multiple fruits, as shown in Figure 1 [19].

Simple fruits are fruits that are formed from a single ovary, containing one or more seeds. Simple fruits can also be further categorized into fleshy or succulent and dry fruits. The pericarp of fleshy fruits is pulpy and soft at maturity, while dry fruits turn hard and leathery. Nuts like the chestnut are a prime example of simple dry fruits, while grapes, bananas, and citrus fruits are examples of simple fleshy fruits [19]. Blackberries and raspberries are examples of aggregate fruits. This category is characterized as fruits that are derived from a single flowering fruit structure (similar to simple fruits) but contain multiple ovaries that can individually contain one or more seeds [19]. Multiple fruits take the definition of aggregate fruits even further, by containing not only multiple ovaries, but including multiple flowering bodies into one fruit component. Pineapples and figs are both multiple fruits and derived from the combination of multiple flowering structures [19]. Fruits can also be classified as climacteric and non-climacteric [20]. While climacteric fruits such as banana can ripen even after they are picked, non-climacteric fruits such as strawberries do not ripen after they are picked. Freshness of climacteric fruits and/or succulent fruits (e.g., bananas) are related to ripeness and emission of ethylene, whereas freshness of non-climacteric and/or aggregate fruits (e.g., blackberries) are mostly related to time, temperature, and/or spoilage (e.g., pH and color).

Fruits go through different stages of growth that vary depending on the type of environmental requirements of the fruit production. For example, maturation refers to the chemical and physiological changes of the fruit throughout its lifetime. For some fruit to continue to mature, it must still be attached to the parent plant, thus maturation is only possible during the pre-harvest period of the crop. As the fruit matures, many noticeable changes occur, including the reduction in chlorophyll allowing the pericarp to take on the actual fruit color, softening of the pericarp, and full seed development. During maturation, most fruits also begin to lower their acidity levels and develop their sweet taste through accumulation of sucrose, fructose, and glucose. As the fruit continues to mature, its rate of respiration will slowly increase. The cell growth rate of the fruit will be steady or increase during this maturation stage and will eventually slow down as the fruit reaches full maturity [19]. While maturation and ripening both describe processes where the fruit will reach a certain peak in its growth, they represent different attributes. Maturation is defined by the biological growth rate of the fruit, but ripeness refers to the development that causes the fruit to reach a desirable stage for consumption. This stage is often described using characteristics such as color and physical texture. This is also the growth characteristic that describes when the desired fruity aroma and flavor levels are reached. Fruits can ripen while being attached to the plant and can continue to ripen even after being harvested [19]. Therefore, freshness monitoring of fruits will mostly utilize ripeness parameters such as color and pH. Senescence in fruits refers to the stage of growth where the plant exceeds its peak maturity or ripeness and begins the irreversible phases of deterioration that will eventually lead to death. During this stage, the cells break down and the fruit loses many of the characteristics that makes it appealing [19]. The senescence of fruits is associated with the emission of different types of gases such as ethylene. Thus, monitoring of senescence can provide important freshness indication of fruits.

The different processing stages of fruits have a big impact on their quality that requires good handling, storage, and transportation techniques to help limit spoilage and wastage while preserving freshness. Once fruits reach maturity or a desirable stage in their maturity, they are harvested and collected for further processing. Although harvesting and processing techniques may differ from fruit to fruit, the general guiding principles, which are known as post-harvesting procedure, are similar [21]. One of the most important prerequisites to maintain fruits freshness is the post-harvesting practices to ensure that the fruits are harvested at the right level of maturity. Maturity indices such as visual, physical, and chemical indices, are all examples of freshness indicators that are used to judge when a fruit can be harvested. For example, the freshness of apples can be identified by using firmness as a physical index to determine desirable maturity, and mangos can use starch content as a chemical index. Furthermore, the calendar date is also a useful estimation for the best harvesting period [22].

Most fruits, especially climacteric fruits, are harvested before they are ripened, so that they can ripen during storage or transport. Fruits should be harvested carefully without any damage. For example, a small bruise on an apple can result in up to a 400% increase in the rate of moisture loss [21]. Although fruits can be harvested by harvesting machines, most fruits, such as apples, pears, plums, and cherries for the fresh market, are harvested manually, which usually results in better quality harvest [23]. After fruits are harvested, storage temperature and humidity are calibrated so that freshness is preserved with minimum spoilage. Inappropriate storage temperatures may increase respiration and water loss, leading to premature ripening. The temperature coefficient (*Q*_10_) is a measure that is used to show the change in rates of biological processes with every increase in 10 °C [24].

(1) $$Q10\;=\;\frac{rate\;of\;reaction\;at\;T_{1}\;+\;10\;{}^{\circ}C}{rate\;of\;reaction\;at\;T_{1}}$$

For most produce, the *Q*_10_ for respiration rate, where *T*_1_ ranges between 5 °C and 25 °C, is about 2.0 to 2.5. This implies that for every increase of 10 °C in that temperature range, the respiration rate will be doubled from the minimum and reduce the overall shelf-life by half. While storage at cooler temperatures is preferred to slow down ripening, too low temperatures can cause chilling injury to the fruit, leading to physical damage and loss of flavor and aroma. Therefore, it is important that minimum and maximum temperatures per fruit are maintained [24]. Relative humidity of the storage room is also critical. Relative humidity (*RH* %) is a ratio of the actual water vapor density in an environment compared to how much the environment can potentially hold. For most fruits, a desirable relative humidity ranges between 85% to 95% [25], as defined by the following equation for relative humidity [26]:

(2) $$\mathrm{RH}\;\%\;=\;\frac{\;P_{Water\;vapour}\;}{P_{Saturation\;water\;vapour}}\;\times \;100$$

Low humidity can cause moisture loss in fruits affecting physical texture and taste. On the other hand, high humidity can speed up fruit decay and rot. Thus, temperature and humidity management are excellent ways to significantly slow down further aging of harvested fruits. Additional examples of external agents to slow down aging include calcium, chlorine solution, and edible coating. For example, calcium can slow down the aging of fruits and microbial growth. Treatments like chlorine solutions or thermal cleansing treatments can prevent microorganisms attaching to living fruit cells and tissues. Edible coating provides a waxy texture as well as barrier between the fruit and microbial invaders [27]. The above discussion of fruits classification, stages of growth, and harvesting/post-harvesting conditions provides an understanding of fruit freshness parameters. This discussion also helps to quantify and estimate the range, sensitivity, and lifetime assessment of freshness sensors and to optimally choose the best freshness sensor for different types of fruits. For example, fruits that degrade quickly due to temperature and humidity are best monitored with temperature and humidity sensors rather than using a microbial pathogen sensor. Also, fruit physiology, growth stages, and harvesting can provide a bridge between freshness sensors and smart packaging sensors for a more improved monitoring of fruits quality.

## 3. Smart Packaging Systems for Fruit Freshness

**Figure 2:**
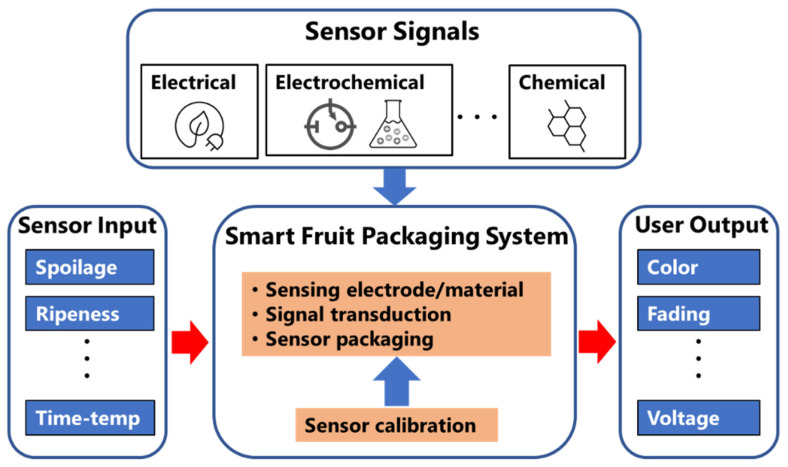
Schematic diagram illustrating the details of a smart fruit packaging system.

Freshness sensors or indicators can sense and inform about the quality of fruits in terms of their freshness level, ripeness, or firmness. Therefore, fruit freshness sensors are on-package indicators that monitors the environment inside or outside the package and allow consumers to make informed decisions about the quality of the fruits [28]. On the other hand, smart packaging and/or sensors refer to an embedded system, which is a combination of electronics and electrochemical/electro-optical sensors that are near packaged fruits, allowing continuous monitoring of the quality of packaged fruits once the package leave the plant until it reaches to consumers, as illustrated in Figure 2. The use of freshness sensors in fruits’ packaging vastly depends on the type of fruits and the fruits physiology. Therefore, a general classification and understanding of the fruit’s physiology is important when designing and implementing freshness sensors and smart packaging systems, as discussed in the following subsections.

### 3.1. Importance of Smart Packaging and Its Relationship with Freshness Sensors 

Fruits packaging is a critical step toward the transport and sale of produce, and it can be categorized into two main types based on its objectives: consumer packaging and transportation packaging [29]. Consumer packaging is the individually packaged collection of fruits that are sold as a single unit, which are usually made of a plastic film or container. On the other hand, transport packaging is designed to allow large quantities of produce to be transported easily, efficiently, and safely. Usually, large plastic, wooden, and cardboard crates are used for transportation; however, plastic containers are preferred because of their high durability and easier sanitation [24]. The primary aim of both types of packaging is to ensure that the products inside are protected from damage and to provide accurate and thorough information about the origin, grade, nutrition etc. of the products within them [29]. The transparency, mechanical stability, and chemical inertness of plastic packaging containers are important aspects to be adapted to integrate smart packaging sensors. This will cause minimal change in fruit packaging industry standards.

Fruits are sold to consumers in a variety of packaging forms. For example, frozen fruits without fat that do not oxidize typically show very long shelf life as compared to their as-harvested counterpart. However, some studies indicated loss of nutrients, such as vitamin C, and distortion of the texture of the fruit when thawed after long-term storage of frozen fruit [30]. Another possible effect of frozen fruit is freezer burn, which is the distortion of taste and texture through dehydration when the frozen food comes in contact with air [31]. On the other hand, canned fruits have an even longer shelf life; however, they contain preservatives and sugars that can take away the nutritional value of the fruit itself compared to a fresh living fruit. Although fresh produce has the shortest shelf life and can easily be the most expensive, particularly if it is of all-natural or organic grade, it is still considered to be most flavorful and nutritionally richer form of produce [32]. For this reason, it is still one of the most popular forms of produce delivery and makes up a large portion of the fruit and vegetable market [32]. Therefore, monitoring fresh fruits with smart packaging is critical for delivering customers with quality, nutritional value, and safety [33]. Thus, the need for better quality monitoring and management systems has become a driving force in the industry. Once the produce has been checked, treated, and left the packaging plants, there is very little to no monitoring that occurs during transport or by the resellers. In most commercial supermarkets, fresh produce is kept refrigerated in storerooms at around 2–4 °C to slow down ripening. While monitoring the storeroom temperature and humidity levels may help slow down decay, some fruits such as bananas that blacken when refrigerated, cannot undergo such growth-inhibiting storage practices. There is also reduced environmental control in the display sections of grocery stores, where produce is left out in the open [34]. The lack of monitoring infrastructure at this point in the processing chain makes it difficult to ensure that the quality of fruits and vegetables is maintained before it reaches the customers. To address these challenges, many companies [35] have started to develop smart packaging technology that can be used to ensure quality monitoring and management from the beginning and throughout the entirety of the process.

While normal packaging practices have been in use since the industrial revolution, the recent innovation of smart packaging systems have made quite an impact in the food industry. In fact, the value of the global smart packaging market is estimated to become USD 26.7 billion by the year 2024 [36]. Smart Packaging describes the embedded systems technology used in product packaging. Traditional commercial packaging does not possess the ability to monitor the quality of the product once it has left the packaging plant. Smart packaging allows us to continue to monitor and maintain quality all the way until the product is consumed. Smart systems can vary from simple self-contained units to fully integrated subsystems interacting together to perform multiple tasks. These specific subsystems can be categorized into the following components: intelligent packaging, data handling and communication, and active packaging. Intelligent packaging refers to the monitoring component of the smart packaging system, where sensors and indicators are used to gauge the quality of the packaged product. Data handling and transmission are also integral to the overall system. Many of the sensors on packages operate independently and can both detect relevant information and display it on the package. However, some smart packaging systems relay sensor data to an external resource for real-time data processing and analysis. Furthermore, smart packaging not only focuses on the monitoring of packaged goods but can also incorporate active elements to mitigate product spoilage for better quality maintenance.

### 3.2. Intelligent Systems in Fruit Packaging 

Smart packaging solutions utilize a variety of sensor technologies to recognize and resolve problems in fruit packaging. Different sensors may be used depending on the type of fruit being monitored. This subsection of the review describes the common freshness sensors used for packaged fruits. 

#### 3.2.1. Direct Freshness 

**Figure 3:**
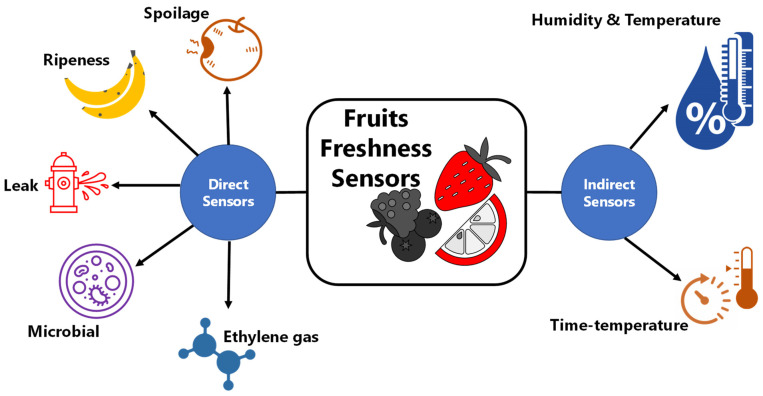
Classifications of sensors for monitoring freshness of fruits.

There are two types of freshness sensors—namely, direct sensors and indirect sensors. As the name suggests, a direct freshness sensor detects a particular analyte directly from the fruit as an indicator for food freshness. On the other hand, an indirect freshness sensor is based on indirect or reaction-based detection of fruits degradation due to certain freshness parameters such as temperature and/or time. These sensors are uniquely designed based on different stages of monitoring such as the food distribution chains and consumer package on the shelf. In any case, the sensors should correctly monitor the rate of deterioration of fruit freshness [37]. An array of concepts for fruit freshness sensors or indicators have been developed including aldehyde [38,39], volatile organic compounds (VOC) [40], ethanol [41], hydrogen sulfide (H_2_S) [42], pH [43,44], and CO_2_ [45]. The sensors or indicators are integrated into the food package as a visible indicator, label, or tag that undergoes color changes according to changes in the freshness markers and/or analytes. Freshness sensors can consist of single, dual, or multiple sensors array. Most commercial freshness sensors utilize a single sensor. Single sensors can only monitor one freshness parameter at a time, such as pH or time-temperature. Dual freshness sensors use two sensors that reference each other in the sensing process while informing food freshness at the same time. However, dual, and multiple sensor array architectures are being developed at the lab scale and will soon be available in the market. Figure 3 shows different types of fruits freshness sensors based direct and indirect sensing methods.

Direct fruit freshness sensors can detect or sense the freshness level of fruits based on unique markers or compounds. Conventional types of direct fruit freshness sensors include spoilage, ripeness, leak, microbial pathogens, ethylene, and senescence indicators. In most cases, the sensors contain a color indicator for easy visualization of freshness level by the naked eye. The indicator undergoes a change of color, and the rate of color change corresponds to the rate of deterioration of the food, which is also correlated with temperature variation and time while the food is in distribution cycles and on the shelf [9].

***Spoilage.*** The spoilage of fruits is caused by the deterioration of their quality to a degree when they are no longer edible. Although spoilage indicators of food may be more related to fish and meats, fruits also show certain spoilage indicators due to different external factors. The spoilage of fruits occurs due to external factors including temperature, humidity, gas, and surrounding atmosphere. Detection of food spoilage can be done using chemical sensors including a pH sensor that perform noninvasive and real-time monitoring of the fruit freshness. The pH of an enclosed fruit package changes within the headspace inside, which can be measured by a pH sensor. Typically, pH indicator dyes are used that undergo color change when put into an acid or base. When a fruit spoils, it releases different types of volatile organic compounds that can be detected with pH sensors. pH sensors provide a cost-effective, highly sensitive, and easy-to-use means for detecting on-package freshness. For example, colorimetric pH indicators for sensing aldehyde have been reported. In one study, a pH sensor used to identify glutaraldehyde in the liquid phase was based on the chemical reaction mechanism of hydrogen bond between glutaraldehyde oligomer created by an aldol reaction and the sensor [39]. In another study, formaldehyde was detected by exploiting the chemical reaction between formaldehyde and primary amines [46]. In both studies, the color change originated from changes in the fruit’s basicity. 

**Figure 4:**
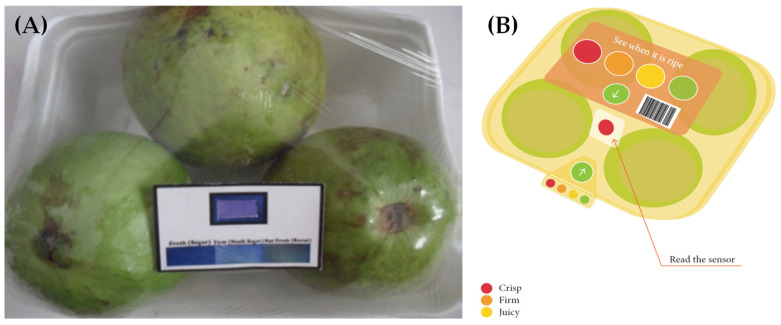
(**A**) Application of the freshness indicator for guavas packaging [49], reproduced with permission from [49]. (**B**) Schematic representation of the RipeSense indicator, reproduced from [35].

***Ripeness.*** An important indicator of fruit freshness is ripeness, which is often difficult for the consumers to estimate. This situation almost always puts the customer in a dilemma about when the fruits are safe to be purchased, stored, or consumed. Several commercial ripeness indicators have been developed. For example, ripeSense^TM^ [47], a New Zealand-–based company, has shown a potential solution to this problem, in which the sensor reacts with the aromas emitted by ripening fruit. The sensor becomes red at the beginning (crisp) and changes to orange (firm) and then finally to yellow (juicy) with increasing ripeness, as shown in Figure 4. Consumers can make informed decisions about the ripeness state of the fruit by simply noting the color of the sensor. The use of this sensor can also reduce fruit damage and shrinkage, which may be caused by the handling and inspection of fruits by the consumers. The sensor package is typically made of recyclable polyethylene terephthalate (PET) clamshell that can provide an increasingly hygienic and environmentally conscious packaging solution. The ripeness of different types of fruits including pears, kiwifruit, melon, mango, and avocado can be monitored with this sensor. Moreover, different stages of fruit ripeness may also release volatile compounds that have been studied using e-noses. One such example is the use of an e-nose for the ripeness determination of tomatoes [48].

**Figure 5:**
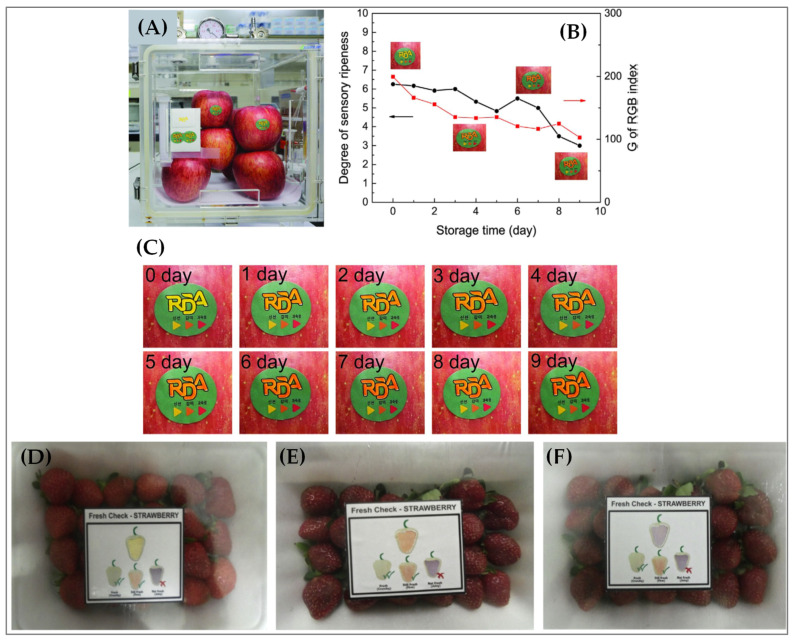
(**A**) Reaction setup for the detection of apple flavor using a sensor label, (**B**) degree of sensory ripeness and RGB index plot vs storage time, and (**C**) color changes in sensor label after exposure to apple flavors, reproduced with permission from ref. [38]. Application of color ripeness indicator for strawberry, (**D**) yellow for fresh (crunchy), (**E**) orange for medium (firm), and (**F**) purple for not fresh (juicy), reproduced from ref. [50].

Secondary species such as aldehyde emission is another marker of fruit (e.g., apple) ripeness. Although extensive studies were conducted regarding aldehyde detection and apple flavor analysis, there have been very few studies on colorimetric sensors for fruits ripeness detection using secondary species emission. For example, a colorimetric sensor was developed that utilized pH indicators based on methyl red (MR) to detect aldehyde emission as a marker of apple ripeness, as shown in Figure 5A–C [38]. In another approach, bromophenol blue (BPB) was used as an on-package color indicator to detect guava ripeness [49]. The BPB was immobilized on a bacterial cellulose membrane using a simple absorption method. With an increased guava ripening process, volatile organic acids were slowly emitted into the package headspace, which caused a decrease in pH level. With decreased pH level, the membrane ripeness indicator subsequently changed its color from blue to green, indicating over-ripeness. Moreover, the membrane ripeness color indicator could successfully detect guava ripeness stored at a varying temperature range of 4 °C to 28 °C. In both temperature ranges, the membrane indicators could accurately estimate the changes of several parameters including pH, solids soluble contents, roughness or softness, and sensory assessment that usually characterizes guava ripeness.

In another study, a simple, low-cost, and on-package color indicator was fabricated using methyl red (MR) towards the detection of ripeness of non-climacteric fruits (e.g., strawberries) [50]. In this case, an increase in the pH in the package headspace caused the release of volatile acids, which gradually reduced the MR that was immobilized onto a bacterial cellulose membrane and caused enzymic formation of esters during ripening. The color of the indicator thus changed from yellow to red-purple, indicating an over-ripeness, as demonstrated in Figure 5D–F. A high correlation was observed between the color changes and the strawberry ripeness. Therefore, real-time ripeness monitoring of strawberries was successfully shown with the use of the on-pack color indicator both in ambient and refrigeration temperatures.

**Figure 6:**
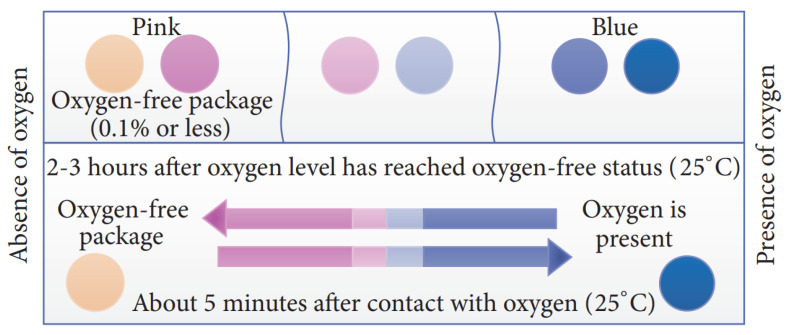
Schematic representation of the leak indicators, reproduced from [35].

***Leak.*** The last section discussed about the ripeness of fruits that indicates the freshness of raw fruits. However, the freshness of processed/packaged fruits is another important parameter. The freshness of packaged fruits can be compromised by the package integrity. Hence, leaks can pose a significant challenge in maintaining the freshness of packaged food. Therefore, leak sensor or indicator can be used to monitor the integrity of packaged fruits that are directly related to fruit freshness. The leak indicators are typically gas indicators that can indicate whether there is any toxic composition of gases produced from the decomposition of packaged fruits endangering consumer health and safety [51]. As shown in Figure 6, changes in the colors of the indicators induced by chemical or enzymatic reactions can then be used to determine the freshness of processed fruits [35].

In many instances of processed fruit packaging, modified atmosphere packaging (MAP) and equilibrium MAP are used, which require gas sensors to indicate fruit freshness [52]. The atmosphere in MAP typically consists of lower O_2_ level (~2%) and higher CO_2_ level (20%–80%). Two major types of MAPs are passive and active MAPs. Passive MAP depends on produce respiration and permeability of the packaging material, which establishes an equilibrium atmosphere inside the MAP. For example, fresh cut apples stored at 2 °C showed that the O_2_ content decreased from 21% (first hour) to 6.89% (190 h), and the CO_2_ content increased from 0.02% to 7.98%; after that, the concentration became almost constant or equilibrium [53]. In contrast, in active MAP, additives are incorporated into the package that help to maintain or extend the product quality. Leak detection sensors (e.g., O2, and CO_2_) can thus monitor the condition of packaged food to give information about the fruit’s quality. Reduced oxygen level in the packaging provides many benefits to fruits. For example, 0.5–5 kPa O_2_ induces reduced ethylene perception and suppressed respiratory and metabolic activity in apples [54]. Hence, any leak in the MAP package considerably increases the O_2_ level that comes from the atmosphere and decreases the CO_2_ level, allowing aerobic microbial growth. Therefore, MAP leak sensors can easily detect the O_2_ level rather than the CO_2_ level [52].

Commercial O_2_-sensitive MAP indicator, for example, AGELESS EYE manufactured by Mitsubishi, uses an O_2_ absorbing sachets [55]. The indicator is placed in direct contact with the gaseous environment surrounding the packaging, which is activated at the time of consumption. When the seal is broken by the consumer, a timer goes off and the indicator color starts changing over time [56]. In another study, a nontoxic surface coating on a substrate was synthesized that activated the irreversible formation of colored spots because of the exposure to molecular oxygen [57]. Another major disadvantage of an O_2_ indicator is the very low sensitivity of the sensor and that it cannot distinguish the presence of residual O_2_ often entrapped in the MAP [58]. The low sensitivity of the O_2_ sensor complicates the handling of the sensor requiring anaerobic conditions during sensor manufacturing and the packaging processes. In addition, the change in the color of the O_2_ gas sensors is not obvious in the presence of acidic CO_2_ gas in MAPs [58,59].

On the other hand, CO_2_ leak indicators do not typically detect leaks in a reliable manner as compared to O_2_ gas sensors. The drawback of the CO_2_ indicators is due to absorption of CO_2_ in the food within the first few days that results in the decrease in the concentration of CO_2_ in the headspace during storage. This applies to only active MAP, in which the package is flushed at the beginning. In contrast, microbial metabolism may result in an increase in the concentration of CO_2_. Therefore, the CO_2_ concentration may still be constant even in the case of leakage and/or microbial spoilage. Commercial CO_2_-sensitive MAP indicators include plastic optical fluorescent films [60]. However, the sensors may suffer from irregular reversibility as the O_2_ entering the package through the leak might be consumed by microbial growth that can lead to the loss of the package integrity. As a result, the color of the indicator may remain the same as that of an intact package even in the case of a food spoilage [60].

***Microbial pathogens.*** Microbial pathogens detection is another important criterion for fruit freshness. In many cases, extraction of a sample is required to be detected with the sensors. Therefore, it is challenging to integrate such sensors within the package that can provide an easily distinguishable change in color at low manufacturing cost. As discussed in the previous section, the easiest way to detect microbial contamination is by measuring changes in gas composition inside the package caused by microbial growth. However, there is a low prevalence of pathogens in fruit packages (Table 1) [61], and there are few reports of package sensors for pathogens detection [62]. Detection of microbial spoilage due to CO_2_ is difficult in a fruit package that already has a high concentration of CO_2_. Therefore, CO_2_ gas detection-–based microbial pathogen sensor may only work in packages that do not contain any CO_2_ gas [63]. Gas-detection-based biosensors were also developed using conducting polymers that may also detect gas released due to microbial metabolism [64,65]. The biosensors were fabricated using conducting polymer nanoparticles inserted into an insulating matrix. The resistance of the matrix changes is correlated with the amount of gas released [66].

Microbial pathogen sensors were fabricated based on microbial metabolites reactions to the enzymes produced by contaminating microbes [67] and the consumption of particular nutrients in the food [68]. These sensors are based on changes of color, which motivated the widely developed optical-based biosensor together with acoustic transduction systems over electrochemical transduction methods. Common pathogens detected in these methods include Staphylococcal enterotoxin A and B; *Salmonella typhimurium*; Salmonella group B, D, and E; and *Escherichia coli* and *E. coli* 0157: H7 [68,69]. Commercial efforts for making biosensors for food packaging include the Food Sentinel System (SIRA Technologies Inc., CA, US) [70] and ToxinGuard (ToxinAlert, ON, Canada) [4]. In the Food Sentinel System, the biosensor can detect food pathogens through specific antibodies attached to the membrane. The membrane was used as part of the biosensor’s barcode. The barcode turned unreadable when the pathogens detected caused localized dark bars. In ToxinGuard, antibodies were printed on polyethylene-based plastic packages as a visual diagnostic system that can detects food pathogens such as *E. coli*, Listeria sp., Salmonella sp., and Campylobacter sp. A comparison of different direct freshness sensors is given in Table 2 below.

#### 3.2.2. Indirect Freshness 

Indirect detection of a fruit freshness detection relies on indirect freshness markers including temperature, storage time, and associated technologies such as RFID (radio frequency identification). Indirect freshness sensors replicate the change of a specific quality parameters of the food facing the same exposure to the indirect freshness markers. The rate of change of the sensors should be correlated with the deterioration rate of the packaged food with the variation of temperature/humidity over time during transportation, distribution, and storage. The indirect sensors should indicate the freshness monitoring results in terms of change of color and electronic signal output when exposed to abnormal storage temperatures and/or humidity. The indirect freshness sensors can be broadly classified as humidity indicators, temperature indicators (TI), time–temperature indicators (TTIs), and RFID-based indicators or sensors.

***Temperature.*** Temperature and humidity inside the fruit package is an important factor to monitor freshness. The freshness of fruit products may rapidly change due to improper temperature and relative humidity conditions throughout transportation and storage. A high relative humidity along with temperature fluctuations can lead to condensation of water. Water condensation can then induce microbial growth such as yeast, mold, fungus, and bacteria. Condensed water droplets may also create blockage in perforated plastic packages resulting in reduced respiration rate and, hence, faster degradation of fruits. Condensed water may also cause fog inside the package, which would impact the customer’s decision to buy the product due to obscure vision of the product. 

**Figure 7:**
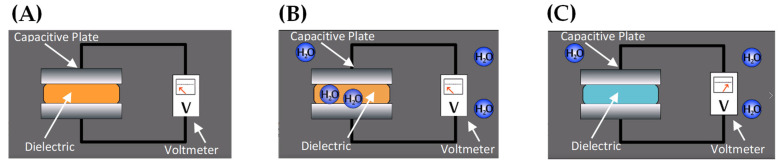
Working principle for a capacitive humidity sensor. (**A**) The initial setup of the capacitive sensor indicating no detectable voltage across the plates. (**B**) The introduction of water vapor in the atmosphere. (**C**) The change in the permittivity of the dielectric because of the water vapor, generating a detectable voltage across the plates.

***Humidity.*** A capacitive humidity sensor is a type of humidity sensor (i.e., hygrometer) that utilizes change in capacitance to detect relative humidity levels. This sensor comprises of a pair of thin metal surfaces or plates known as electrodes separated by a film of dielectric material, such as a metal oxide (Figure 7). These two plates form a parallel plate capacitor with some level of capacitance. As the moisture in the atmosphere fluctuates, the permittivity of the dielectric film also changes. This change results in a change in capacitance between the two plates. The variance in capacitance generates a detectable analogue voltage difference across the two electrodes, which can be measured and used to determine the relative amount of moisture in the environment, according to the following equation:

(3) $$C=\;\frac{k\varepsilon ˳\;A}{d}$$

The capacitance is determined through the above equation, where *C* is capacitance, *A* is the area of one plate, *d* is the distance between the two plates, ε˳ is the permittivity of free space constant (8.85 × 10^−12^ F/m), and *k* is the dielectric constant that changes depending on the characteristics of the dielectric material, as given by the following equation:

(4) $$V=\;\frac{Q}{C}$$

Voltage can be determined by capacitance through the relationship above where *V* is voltage, *Q* is the amount of charge on a plate, and *C* is the capacitance. One of the most beneficial features of these capacitive humidity sensors is that their sensing range for relative humidity spans from 0% to the full 100%. This key specification must also be considered with the accuracy specification, which in general is about +/− 2% relative humidity. The operating temperatures for these sensing devices also incorporate the range in temperature values of −20 °C to 85 °C that are encountered during fruit transport and storage [26]. While these sensors provide great range and are some of the most accurate humidity sensors on the market, they are expensive and suffer from long-term stability problems. Therefore, it is important to recalibrate or replace these sensors once they have reached their recommended life span, especially when reusing the same sensor for different batches of produce for transportation packaging. Moreover, capacitive humidity sensor performance may be limited due to problems related to saturation, hysteresis or exposure to prolonged high humidity conditions.

***Time–temperature.*** Time–temperature indicators (TTIs) are also indirect freshness sensors that work based on different chemical, physical, and biological mechanisms [77]. The chemical and physical mechanisms are based on a chemical reaction or physical change, respectively, due to changes in time and temperature. Examples of such mechanisms include acid–base reactions, melting, and polymerization. Biological mechanism is, on the other hand, based on changes in the biological activity. Examples of biological activity include microorganisms, spores, or enzymes due to changes in time and temperature. TTI sensors are mostly based on change of colors when the sensors are exposed to higher than recommended storage temperatures for extended period, and they also signify changes when the product reaches the end of its shelf life. Therefore, physical, chemical, and biological activity in fruits can be monitored using TTIs that can give a clear, accurate indication of the freshness of fruits in terms of their quality, safety, and shelf life.

Irrelevant to the method of detection, some key specifications that all TTI datasheets should include are threshold temperatures and runout times. As the name suggests, the threshold temperatures specify the operating range for the indicator, i.e., the maximum and minimum temperature values that need to be exceeded for the indicator to start recording. The runout time refers to the minimum amount of time required at a temperature outside the operating range for the entire indicator to have changed color. While these indicators do give a relatively good time estimate of exposure to non-desirable temperatures, they are not as accurate as conventional temperature sensing devices. However, their simple operating principle, ease of use, and the fact that most customers do not require exact measurements makes this an ideal sensor to be used for commercial fruit smart packing systems [78].

**Figure 8:**
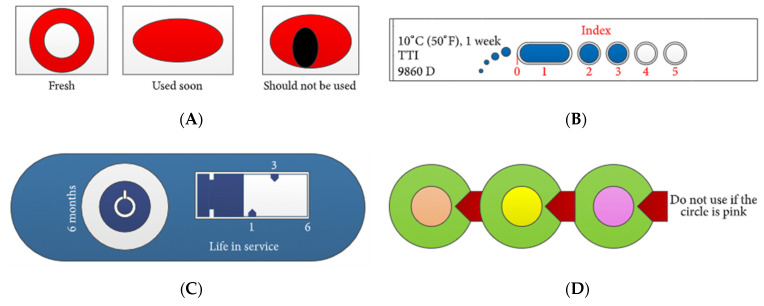
Schematic representation of the TTI, (**A**) Fresh-Check, (**B**) Timestrip, (**C**) MonitorMark, (**D**) CheckPoint, redrawn and reproduced with permission from [35].

Many commercial TTIs has been developed that are widely used for monitoring perishable goods. Examples of such TTIs include Fresh-Check^®^, Monitor Mark^TM^, OnVu^TM^, eO^®^, Timestrip^®^, Checkpoint^®^, and Tempix^®^, as illustrated in Figure 8 [37]. These commercial indicators are mostly based on chemical and enzymatic reactions and, in most cases, can be used for fruit packages. For example, Fresh-Check^®^ is a polymer-based self-adhesive chemical indicator with visual color change, which is a full history type sensor based on a solid-state polymerization reaction. Monitor Mark^TM^ is an example of a partial-history TTI that provides temperature vs. time history of the product and is based on the diffusion of blue-dyed fatty acid ester. The OnVu^TM^ indicator is based on photochemical reaction that indicates whether the product was exposed to high temperatures over time. eO^®^ is a microbial-based TTI that changes its color due to changes in pH of the degraded food products. Timestrip^®^ is a diffusion-based TTI in which the dye melts and migrates through the porous membrane of the indicator when the temperature is higher than the reference temperature. Checkpoint^®^ indicator is an enzyme-based TTI that changes its color due to enzymatic reaction. Tempix^®^ is another example of diffusion-based TTI in which activation liquid diffuses into a barcode in case of any breach of temperatures. Due to diverse mechanisms of the commercial TTIs, they can be used for freshness detection of different types of fruits based on their degradation mechanism with the breach of time-temperature. A comparison of different indirect sensor systems is given in Table 3.

### 3.3. Intelligent Packaging System in Container 

***RFID-based sensor system.*** Most of the previous sensing systems discussed focus on the quality of the fruits or fruit products, but there is still significant data that can be collected from the fruit container itself. Intelligent packaging can also make use of radio frequency identification device (RFID) technology to track and identify the product quality during storage and transport in containers. RFID tags are electronic barcodes used to identify individually packaged units whose data can be stored on a network and accessed later as needed. These systems require an RFID tag with an antenna, an RFID scanner, and utilize radio waves to communicate data to a network. The tag itself is an IC with an antenna, held together by a protective covering. When scanned, the RFID tag relays information about the package through the antenna to the reader, which has a receiver that transforms the radio waves emitted by the tag into the appropriate data format [79]. These scanners can either be isolated units or can be connected to a central network that can store, processes, and further propagate the received data as designed. 

RFID tags themselves are categorized into either passive or active tags. The main difference between these two types of tags is their power source, as RFID tags require power to function. Passive tags have no internal power source and thus rely on the electromagnetic energy emitted by the reader, whereas active tags have their own on chip batteries for power. By being self-powered, active tags allow for real time traceability as they can constantly send out information. While active tags do provide this additional functionality, their high cost may make them less favorable in some situations, in comparison to the passive variety [80]. 

**Figure 9:**
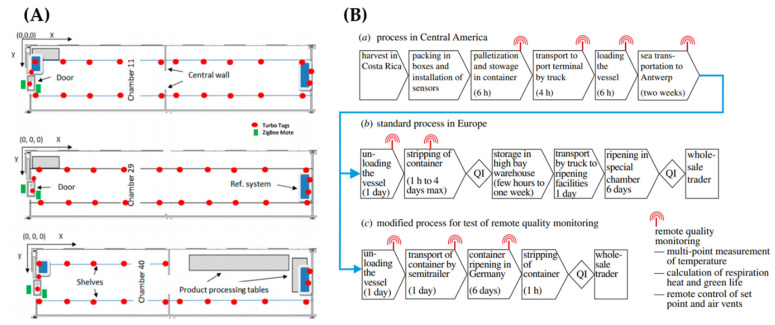
(**A**) Schematic diagram of the cold rooms and sensor distribution [81]. (**B**) Simplified structure of the cold chain for bananas, from harvest to ripening by ethylene treatment, with regular duration of steps, reproduced from [85].

Temperature and humidity of refrigerated fruit storage was developed by two wireless sensing technologies, RFID and wireless sensor network (WSN), as illustrated in Figure 9A [81]. In this study, a combination of RFID and WSN devices were used in three commercial wholesale chambers with different set points and products. A set of 90 semi-passive RFID temperature loggers were used to record a 3D temperature mapping and psychrometric data modelling that calculated the changes in enthalpy and the absolute water content in air. The combination of the RFID and WSN sensor network enabled the estimation of energy consumption in the cold storage, water loss from the products as well as detection water condensation over the stored commodities.

RFID technology provides many benefits in packaging applications. Inventory can be easily monitored using RFID readers placed at the entrances of the storage location. Although RFID sensors have a limited reading range, they do not require a direct line of vision or physical contact with the scanners. Therefore, detection of these tags is very simple when the fruits are within a carrier container or storage shelf. Moreover, the RFID reading range can be adjusted based on the operation frequency and power supply circuit unit. This is a great advantage over traditional and less durable barcode technology that requires unobstructed line of sight with proper orientation to be read. Additionally, RFID scanners can read multiple tags at the same time and allow for both single item and bulk scanning. While RFID tags have many advantages over conventional barcode labels, they have several disadvantages. For example, RFID tags can be prone to interference, particularly when the package is surrounded by materials such as metal that can interfere with the radio waves. The cost of installing a RFID based system is also much higher than conventional barcode technology, which is an important factor for mass production. Using RFID tags for reusable containers or packages can be a cost-effective way of being able to reap the benefits of the technology without substantial financial strain [82]. Freshness sensors are being employed commercially with RFID tags to monitor relative humidity, temperature, light exposure, pressure, and pH of foods. RFID-based sensors can identify any possible interruptions of the cold chain that may undermine food safety and quality [83]. For example, a pH sensor embedded in a passive RFID tag was developed for in situ monitoring of the deterioration processes of packaged food [83]. Therefore, RFID-based sensors have tremendous potential in wireless food quality monitoring at low cost.

In another study, real-time temperature and humidity monitoring of a small cold storage of fruit and vegetable were demonstrated using an Arduino microcontroller-based temperature and humidity monitoring system [84]. The system hardware components included microcontroller unit, wireless communication protocols, temperature and humidity sensor, and organic light emitting diode (OLED) display. The system software components included Arduino IDE, UartAssist serial debugging assistant, and Lighting Blinker. The system test results showed that it can perform remote monitoring with high measurement accuracy and ease-of use. In a case study of remote quality monitoring in the banana chain, the transport of bananas from Costa Rica to Europe were modelled and validated, as shown in Figure 9B [85]. It was also showed that this model can be applied to generate automated warning messages for containers with temperature and humidity variation that would allow for remote monitoring of the ripening process inside the container.

**Figure 10:**
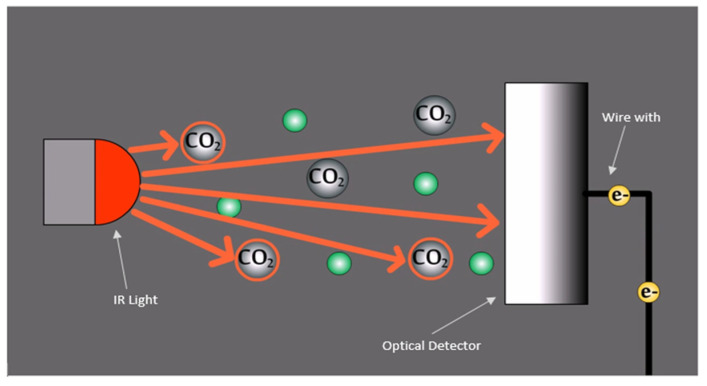
Setup of the non-dispersive infrared (NDIR) sensor. The infrared (IR) light with a wavelength of 700 nm to 1 µm is partially absorbed by carbon dioxide and partially transmitted through to the detector.

***Carbon dioxide non-dispersive infrared (NDIR) sensor.*** Consumer packaging with modified atmospheric environment helps to ensure higher levels of carbon dioxide and low levels of oxygen within the package. Compromising the integrity of the package can lead to early decay in the fruit and the possibility of microbial infection. A high oxygen level accelerates the ripening of fruits as well as the growth of microbial organisms [9]. In contrast, a low oxygen atmosphere slows down respiration and prevents the early ripening or fruit death. However, too low levels of oxygen can cause cell death leading to premature decay. Thus, the monitoring of atmospheric gases with a technique such as non-dispersive infrared (NDIR) sensors can help to ensure proper packaging conditions. A NDIR sensor uses infrared light absorption to determine the concentration of specific gases. Typically, light with wavelengths from 700 nm to 1 µm is shone through the air that contains the target gas to an optical detector. The atmospheric gases absorb certain wavelengths of light, while permitting others to pass through. Consequently, the amount of target gas particles that are present are measured by the amount of light absorbed, which is sensed by the optical detector that converts it into a measurable electrical output [72]. NDIR sensors make use of infrared technology to determine the concentration of specific gases in the atmosphere by measuring the light absorption characteristics. This is illustrated in Figure 10. Different gases can absorb different wavelengths of light while permitting others to pass through. Using this property, the amount of target gas particles that are present can be related to the amount of light absorbed or, conversely, the amount of light still able to be passed through and sensed by the optical detector. The amount of light sensed at the optical detector is then converted into a measurable electrical output [9,83,84].

Smart packaging systems for fruits can use carbon dioxide as the target gas for transport containers of large volumes of produce with a controlled atmospheric composition. In case of any broken packaging seal, carbon dioxide and oxygen levels are disturbed, triggering seeping of external oxygen and dissipation of carbon dioxide, accelerating the respiration rate of fruit ripening. Carbon dioxide can be easily detected with NDIR sensors at an infrared wavelength of about 4300 nm, as this is not absorbed by oxygen. Important parameters of NDIR sensors in fruit packaging include measuring range, sensitivity, and accuracy. The measuring range refers to the percentage of air that is carbon dioxide. Standard NDIR sensors can support a range of 0%–20% Carbon dioxide detection and can be measured with an accuracy of +/− 0.5% and a sensitivity of about 0.05% [86]. Other advantages include long lifetimes, as the same sensor can be reused in multiple fruit shipments, and minimal maintenance required. However, the optical detector can be prone to external spectral interference, leading to inaccurate light detection, which can be avoided by using optical filters [87]. A major drawback of NDIR sensing for smart packaging is that it is more suitable for system level smart packaging where the NDIR sensor is located outside the food package and provides feedback to system actuators to improve storage conditions.

***Ethylene.*** Phytohormones are naturally occurring plant chemicals that help to facilitate and regulate development and maturation in plants. Ethylene is a prime example of such a chemical. Ethylene is a plant hormone that can accelerate the ripening process in climacteric fruits, like tomatoes, bananas, and apples, through the degradation of starch and the formation of sugars [88]. Usually, ethylene is emitted in higher concentration from climacteric fruits compared to that of non-climacteric fruits [89]. Found in plant tissues, ethylene is a key component that helps regulate the ripening progression of fruits and is correlated with the respiration of fruit [90]. Fruits continue to live and respire even after harvest, consuming stored carbohydrates (in the form of glucose) and oxygen to produce water, carbon dioxide and energy to carry on life processes, as represented by the following equation [90]:

C_6_H_12_O_2_ + 6 O_2_ → energy + 6 CO_2_ + 6 H_2_O (5)

Ethylene is released by fruits to trigger respiration so that energy can be produced for internal biochemical processes. As this continues, the flavor, texture, and nutrition of the plant also changes; thus, a continually high pace of respiration can cause the fruit to ripen quickly and eventually start decaying. Not all fruits ripen after harvest; some fruits go directly to the senescence stage. Fruits such as bananas are extremely sensitive to external ethylene as well because of its climacteric property. Hence, it can be noticed that when one banana in a bunch begins to ripen, the others in close proximity will start to ripen as well [91]. It is evident that the amount of ethylene present must be supervised in order to prevent the fast degradation of fruit [92].

**Figure 11:**
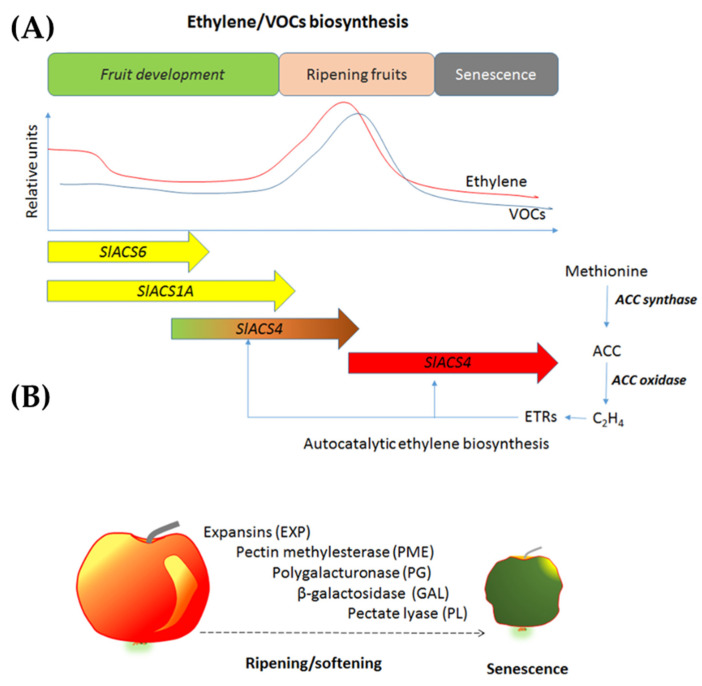
(**A**) Schematic and simplified biosynthesis of ethylene and VOCs in fruit development. VOCs biosynthesis originate from different pathways, for example phenylpropanoids, fatty acid, and carotenoids degradation. (**B**) The main enzymes participating in cell wall degradation during fruit ripening and senescence, reproduced from [92].

Therefore, ethylene is commercially used to artificially control fruit ripening in storage facilities. Fruits with different stages of ripening that are stored close to each other may also cause a decreased lifespan due to the excretion of ethylene from ripe fruits [93]. Thus, scavenging and monitoring of ethylene is highly recommended to maintain fruit freshness. As explained in Figure 11, ethylene scavenging helps to lower the loss of fruit products due to overproduced ethylene. Typically, potassium permanganate (KMnO4) is used as an ethylene scavenger, which oxidizes ethylene to ethylene glycol. Moreover, ethylene glycol can also be further oxidized to CO_2_ and H_2_O, which results in dark brown MnO_2_ [40]. Commercial ethylene scavengers include KMnO4 granules over clays or activated carbon [94] and low temperature oxidation over a platinum catalyst on mesoporous silica that can remove 50 ppm ethylene at 0 °C [95]. 

**Figure 12:**
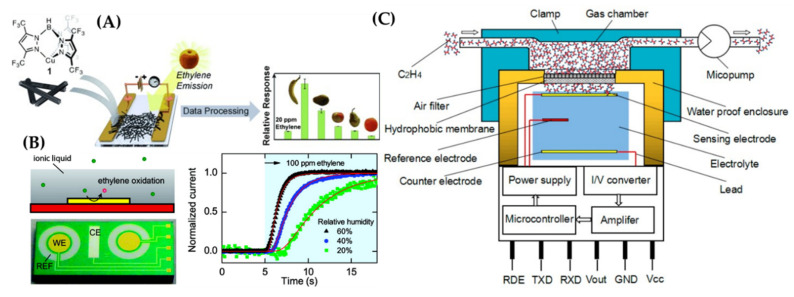
(**A**) Schematic diagram of an ethylene chemoresistive sensor, reproduced with permission from [73]. (**B**) Schematic diagram of the ethylene sensing mechanism, reproduced with permission from ref. [74]. (**C**) Schematic diagram of a smart electrochemical ethylene sensor, reproduced from [75].

The use of ethylene sensors is an alternative way to control fruit freshness by detecting quick ripening before fruit degradation. Ethylene sensors were developed using chemoresistance and electrochemical methods. A chemoresistive sensor was fabricated using single-walled carbon nanotubes (SWNTs) mixed with a Cu (I) complex, which was positioned between gold electrodes [73]. The resistance of the sensor would change upon binding to ethylene (Figure 12A). The sensor had high performance in the presence of interfering species such as acetonitrile and tetrahydrofuran. In another study, an electrochemical ethylene sensor was developed with a thin layer of ionic liquid as electrolyte, as shown in Figure 12B [74]. A gold working electrode was used to oxidize ethylene in a potential window starting at ∼600 mV. The amplitude of the sensor’s output current response time was dependent on the thickness of the ionic-liquid film, relative humidity, and applied potential, which were theoretically modelled based on diffusion processes. In another electrochemical ethylene sensing system, a micropump and a signal conditioning circuit were implemented, which resulted in a rapid detection of ethylene down to 0.1 ppm in air within 50 s [75], as depicted in Figure 12C. Three types of fruits—apples, pears, and kiwis—were used in this study to detect trace ethylene in air exhaled by fruits at low concentrations of under 0.8 ppm. The use of electrochemical methods, therefore, could be a promising approach for electrochemical gas sensors in fruit packaging applications. A comparison of different intelligent systems in fruit packaging container is given in Table 4.

### 3.4. Active Systems in Fruit Packaging

Active packaging takes the technology of intelligent packaging a step further. The idea is to provide a continuous method of quality management to each individually wrapped package. Through regulatory components embedded into the packaging material, the package itself can release or absorb substances and adjust internal atmospheric conditions in order to respond to the needs of the packaged product. Sensors will detect unfavorable elements in the package environment and can react accordingly to mitigate it. This is ideal for extending the shelf life of perishable items even when they cannot be in ideal storage situations [96].

***Ethylene scavengers for active packaging.*** While the fruit is still in transport or storage, it is advisable to keep the amount of ethylene as low as possible. Once higher than acceptable amounts are detected by the sensors, ethylene scavengers can be released to mitigate the effects that ethylene would normally have. Scavengers are compounds that react with the target compound in order to absorb or mutate it into a harmless compound. In the case of ethylene, potassium permanganate is an ideal choice, as it oxides ethylene through stages, with a net result of carbon dioxide, water, and manganese dioxide [40].

***Carbon dioxide emitter for active packaging.*** Opposite to the ethylene scavengers used in the active packaging technology to lower the amount of ethylene gas that fruits are exposed to, carbon dioxide emitters increase the amount of compound, specifically carbon dioxide, present in the package. An example of how this technology is implemented is by utilizing the reaction between sodium bicarbonate and water, which results in the production of carbon dioxide to slow down fruit respiration.

***Moisture absorbers for humidity control.*** Moisture absorbers are compounds that are generally packaged with the product and are used to control humidity levels. These compounds absorb the excess moisture in the packaging atmosphere, resulting in a more controlled environment. Silica gels, which have been shown to absorb water equaling 35% of their own weight, and calcium oxide are examples of such compounds used in packaging dry foods like fresh fruit [97].

***Hydrogen sulfide fumigation for active packaging***. There is compelling evidence regarding the use of hydrogen sulfide (H_2_S) on physiological processes in plants, including seed germination, root organogenesis, abiotic stress tolerance, and senescence of cut flowers. However, the use of H_2_S in regulating ripening and senescence of postharvest fruits is becoming an emerging technique. For example, in one study, the effect of H_2_S on postharvest shelf life and antioxidant metabolism in strawberry fruits was investigated [42]. It was found that a dose-dependent fumigation with H_2_S prolonged the postharvest shelf life of strawberry fruits. Different concentrations of H_2_S were used to fumigate strawberries, and the results showed significantly lower rot index, higher fruit firmness, lower respiration intensity, and polygalacturonase activities than the controls. The study also revealed that higher activities of catalase, guaiacol peroxidase, ascorbate peroxidase, and glutathione reductase and lower activities of lipoxygenase was achieved with H_2_S treatment as compared to untreated controls. Moreover, H_2_S helped in reducing malondialdehyde, hydrogen peroxide, and superoxide anion to lower levels as compared to control fruits during storage. These results indicate that H_2_S can play an antioxidative role in prolonging postharvest shelf life of strawberry fruits, and potentially other similar fruits. A comprehensive list of commercially available intelligent packaging and active packaging systems and their comparison are given in Table 5 [98].

## 4. Challenges and Prospects

Current smart packaging technology has really set a solid foundational basis from which even more food-related processing can take place. While there is great potential for future applications, there are some challenges that may arise because of further development of this technology. The purpose of this section is to discuss some of these challenges and the prospects for food smart packaging. 

***Improvement of Sensor Technology.*** One of the main limiting factors for the further development of smart packaging is the limitation in sensory technology. While there are many sensors that could give accurate readings for different freshness parameters observed in produce and other food products, their usability, specifically size and cost, makes it difficult to incorporate them in large-scale packaged containers. 

The balance between functionality, portability, and size has always been an area of interest in technology. This problem is even more pronounced in the food industry, as there is a push toward good quality foods with lower costs and high availability. One solution to this may be the further development of nanotechnology research in sensors. These sensors can be used to detect air emissions, temperature, humidity, and a variety of other environmental parameters. They also can perform more invasive measurements of food quality, without considerable damage to the product. While this type of technology is not yet economically viable for mass produced food packaging, with further research, it can be commercialized for highly accurate food quality sensing [27].

***Temperature Cross-Sensitivity.*** Another important factor limiting the performance of freshness sensors is the effect of temperature cross-sensitivity. While some freshness sensors are intended to be temperature-sensitive and provide temperature-dependent freshness status of the fruit (e.g., time-temperature, microbial), some other sensors should not be affected by changes in temperature (e.g., electrochemical pH or ethylene sensors). Also, the activation energy or *Q*_10_-factor of a chemical process in any fruit should match with TTIs responses to provide accurate time–temperature profiles. Therefore, proper design considerations need to be taken when developing TTI and other sensors, which sometimes may require fruit-specific sensor design.

***Improvement to Active Packaging Technology.*** One of the main motivations in improving food quality sensing is to be able to implement spoilage inhibitors through active packaging technology. An area of improvement in this research field is antimicrobial packaging. Food sanitization requires the removal of microbial organisms in order to prevent damage to the product. Current antimicrobial packaging systems generally use readily available inorganic compounds. The use of artificial chemical components in food processing is a possible safety concern, particularly in the case of unknown possible reactions or long-term effects. Therefore, these systems can be greatly improved by incorporating naturally derived bio-active agents [41].

***Waste Management.*** Smart packaging consists of different technology embedded into one system. The monitoring systems and smart packaging systems, while necessary for food monitoring, pose problems for the disposal stage of the packaging. Most of the material used is neither biodegradable nor easily recyclable—indeed, some forms of packaging that utilize specific chemical compounds need to be carefully regulated during disposal. Research is currently being conducted in biodegradable or compostable materials for use in such technology, and while not all components can be completely replaced, those that can be substituted will significantly reduce the environmental impact of such systems [41]. 

***Real-Time Control and Data Processing.*** The smart packaging technologies discussed in this review mainly involve self-contained sensor and active packaging technology that can perform on-site processing. New and novel smart packaging technology could potentially make use of the concept of the Internet of Things (IoT) to communicate with external processing units. IoT systems use networks to communicate and share data between multiple components of a system. IoT infrastructure expands on the idea of external communication that RFID systems offer by incorporating two-way data transmission between the “tag” and “reader” components. In this way, the package circuitry could be controlled by an external unit.

The analysis of the data collected by the sensing systems is also a prospective area of growth in the food industry [42]. Upcoming smart packaging systems have the capacity to make use of powerful external computing units that have much higher performance capabilities and processing power than conventional RFID readers. Sensing devices in packaging would collect data and then transmit it to these peripheral computing units for analysis. The information that can be extracted from this data would aid in forming predictions about the patterns of food spoilage and the most effective combination of active packaging technologies for each individual product. Not only can data be sent to industrialized computers, but with the incorporation of IoT technology, it could be used in consumer-oriented applications. Apps that run on mobile devices can be used to track food quality and environmental conditions and recommend storage strategies at home or relay detailed package and product data for in-store use.

**Figure 13:**
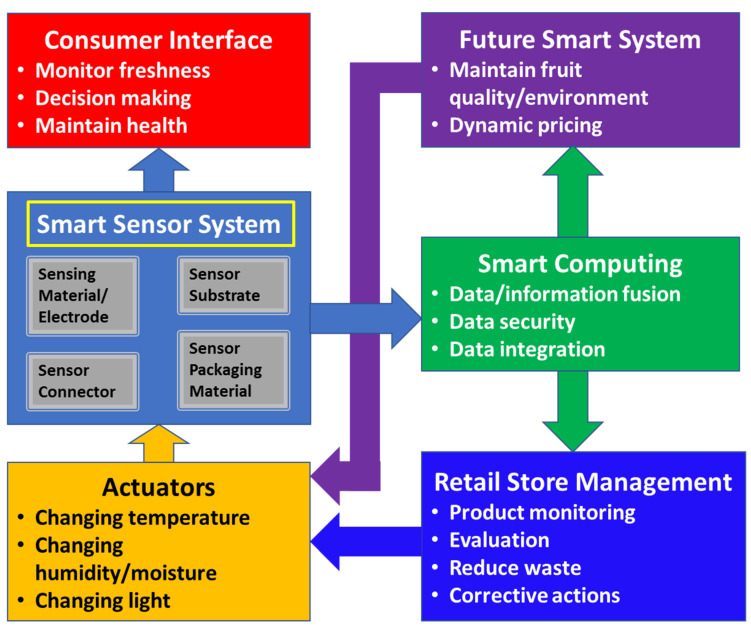
Schematic diagram illustrating how Smart Sensor Systems can be integrated with smart computing, and consumer and retailer needs for monitoring fruit quality, reducing waste, and improving consumer well-being.

Another possible future direction of smart sensor systems is their integration with smart computing and consumer and retailer needs. These integrated systems will enable improved monitoring of fruit quality, thus reducing waste and improving consumer well-being, as illustrated in Figure 13. For example, an emerging smart sensor system should be easy-to-read and interpret, low-cost, biocompatible, green, compatible with existing packaging systems, and capable of multi-sensor fusion for improved monitoring. One outcome can be dynamic pricing of fruits that allows customers to take advantage of, for example, quality versus price. Future integrated smart packaging systems will also benefit our environment, being green and reducing spoilage and waste. For example, such systems will allow for prolonging the life of fruits, help in maintaining better fruit conditions for consumers and retailers, and reducing waste during transport and shelf-life.

While there is great promise in the consolidation of intelligent sensing systems and external processing networks, the issue of cybersecurity is a real threat today. Any infrastructure that relies on communication between two or more separate peripherals has the potential for infiltration and data leakage. Depending on the sensitivity of the collected data, this could pose a serious problem to the advancement of network-based food-packaging systems. Establishments would have to strive to implement best security practices and adhere to strict privacy standards and policies. As an example, Walmart and IBM have partnered to implement blockchain based solution for Walmart to automate their food supply chain procedures. This is to improve traceability, accuracy and achieve a tamper-proof system throughout their company processes [43].

## 5. Conclusions

Current smart packaging systems along with embedded sensor and communication technologies can provide means of monitoring and regulating the quality of packaged fruits. Active packaging technologies extend these intelligent sensing systems by providing a mechanism to maintain food quality and mitigate any harmful environmental elements. Smart packaging technology can be used in a wide variety of food-related industries, including fresh produce. The nutritional value of fresh fruits and vegetables, along with its consumer popularity over frozen or canned alternatives, provides incentive for further development of quality sensing technology in this field. This technology can benefit the global food market by reducing the amount of preventable food spoilage that contributes to wasted resources. Specialized sensors can be used to detect observable characteristics of fruits and vegetables as they ripen and decay. By being able to monitor these features, actions can be taken to prolong the freshness and life span of perishable items in non-intrusive ways.

The emergence of smart systems in food processing applications is still recent and has many more years before it can be considered a routine practice in the industry. While the concept provides a novel way of ensuring the highest quality of food, there are still many obstacles that need to be addressed. Waste management during the disposal of smart packing systems poses environmental and economic complications, along with the safety concerns associated with using non-biological chemicals and compounds in spoilage mitigation techniques. The progress of sensor technology itself is also a restriction on the effectiveness of this application. Despite these challenges, there is huge promise in the prospects of this technology. Innovations like nanotechnology, natural-derived active packaging agents, and biodegradable components are examples of solutions currently under development to address these challenges. The utilization of IoT networks with embedded sensor technology and mobile processing also has a promising potential. By ensuring the issues of security and data privacy are properly addressed, these types of solutions could provide significant gains to the entire food industry. With the pursuit of continued research in this field, the day may not be far away where we see such technology in everyday food related applications.

In summary, we reviewed the current state-of-the-art of freshness monitoring and smart packaging technologies for fruits quality. These technologies provide promising solutions towards conventional packaging such as loss, damages, and wastes. The biology of fruits, their classifications, growth and different stages of processing and harvesting were discussed. This background information was analyzed due to the need for smart packaging that can help reduce fruits wastes during harvesting, pos-harvesting, and packaging stages. Important freshness indicators for fruits and packaged fruits were discussed, such as spoilage, ripeness, leak, microbial pathogens, ethylene response, temperature, humidity, and time–temperature. Monitoring these parameters was discussed through intelligent systems such as electrochemical sensors, optical sensors, capacitive indicators (TTI, gas and humidity), and electronic system (RFID tags). Also, adjustment these parameters were discussed in terms of active packaging such as using scavenging objects (oxygen or carbon dioxide scavengers, moisture absorbers), or releasing objects (hydrogen sulfide). Challenges of the technology faced by the industry was discussed in terms of complexity of fabrication, cost, regulation, and life cycle sustainability of the sensors and systems. A cross-discipline collaboration between the industries, academia, and the consumers may provide more sustainable solutions in the development of smart packaging systems for fruits quality monitoring.

## Figures and Tables

**Table 1:** Pathogen prevalence in fruits, median prevalence and country of prevalence, reproduced from [61].

Pathogens	Fruits	Median (Range)	Sample Country
*Escherichia coli* O157:H7	Apple Cantaloupe Fruit (not articulated) Peach Raspberry	0 (0) 1.7 (0–40.0) 0 (0–4.00) 2.5 (0–10.0) 0 (0–40.0)	United States, Mexico, Germany, South Africa, United States
Verotoxin-producing *E. coli*	Citrus	0 (0)	Japan, United States
Pathogenic *E. coli*	Kiwifruit	0.50 (0–3.9)	China
*Salmonella*	Apple Cantaloupe Citrus Fruit (not articulated) Kiwifruit Peach Raspberry Strawberry	0 (0) 1.7 (0–40.0) 0 (0) 0 (0–4.00) 0.50 (0–3.9) 2.5 (0–10.0) 0 (0–40.0) 0 (0–30.0)	United States Mexico, Japan, Germany, South Africa, China, Poland, Poland, Spain
*Listeria*	Blackberry Blueberry Cantaloupe Fruit (not articulated) Peach Raspberry Strawberry	0 0 1.7 (0–40.0) 0 (0–4.00) 2.5 (0–10.0) 0 (0–40.0) 0 (0–30.0)	Poland, Mexico, United States, Germany, South Africa, Spain
*Staphylococcus aureus*	Kiwifruit	0.50 (0–3.9)	China
Norovirus	Raspberry	0 (0–40.0)	Poland

**Table 2:** Comparison between different direct freshness sensors.

Freshness Parameter	Smart Systems/Sensor	Main Components	Response	Advantages	Disadvantages	Reference
Spoilage	pH chemical sensors	pH indicator dyes	Color change due to pH increase	Cost-effective High sensitivity Easy-to-use	No quantitative data Chemical migration to food	[50,71]
Ripeness	Aromas sensors, e-noses sensors, pH chemical sensors	Electrical circuits pH indicator dyes	Absorption of volatile organic compound leads to physical change on surface sensor Color change due to pH increase	Very accurate Can be used in bulk packaging	Expensive	[48,50,71]
Leak	O_2_ sensors, CO_2_ sensors	Plastic optical fluorescent films Infrared Red technology	Color change in the films Light absorption measurement	Integrated into packages May be checked by eye or optical devices	Low sensitivity CO_2_ absorption by food Spectral interference	[60,72]
Microbial	Microbial metabolites based, Gas detection based, Optical based biosensors	Antibody specific membranes, Optical fluorescent films	Color change due to reactions with metabolites	Low cost. Can be used in headspace.	Possible migration of chemicals to food Sensitivity depends on transducer and quality of antibody	[67,68,69]
Ethylene	Chemoresistance sensors, Electrochemical sensors	Electrical circuits	Resistance changes Current changes	Provide quantitative information Good repeatability and accuracy	Cross sensitivity with gases Sensitivity to temperature and humidity	[73,74,75,76]

**Table 3:** Comparison between different indirect freshness sensor systems.

Freshness Parameter	Smart System/Sensor	Main Components	Response	Advantages	Disadvantages	Reference
Humidity	Capacitive sensors	Electrical circuits with capacitive electrodes	Change in capacitance is translated to voltage change	Accurate Provide quantitative /qualitative information	Expensive Cross-sensitivity	[26]
Time-Temperature	Polymeric based, Photochemical based, Microbial based, Diffusion based, Electronic based, Enzymatic based	Detect quality of package environment and fruit produce Detect the passing of threshold limits of different factors (temperature, gas composition disruption)	Irreversible color change due to exposure to above threshold temperature over period	Provide visual information Low cost Placed outside the package	No information about food quality	[37]

**Table 4:** Comparison summary of intelligent systems in fruit packaging container.

Technology	Purpose	Advantages	Disadvantages
Electrochemical Ethylene Sensor	Detect quality of fruit produce Detect concentrations of ethylene released from fruit Determine rate of respiration and ripening in fruits	Fairly sensitive to ethylene Can be used in individual and bulk packaging Less expensive than NDIR	Can be subjected to chemical interference (can be internally mitigated) More expensive than indicators
NDIR	Detect quality of package environment Determine concentrations of gas in atmosphere Detect leakage or disruptions in atmospheric conditions	Very accurate Can be used in bulk packaging	Expensive Not practical in individual packaging
Capacitive Humidity Sensor	Detect quality of package environment Detect relative humidity of package	Very accurate Can be used in individual and bulk packaging	Expensive compared to other humidity sensors Low long-term stability
Indicators (TTI, Freshness, Integrity)	Detect quality of package environment and fruit produce Detect the passing of threshold limits of different factors (temperature, gas composition disruption)	Easy to read Easy to incorporate into packaging Inexpensive	No quantitative data Not as specific as sensors
RFID	Identify and relay package information Relay package information to external processing unit	Scalable Real-time monitoring Easy to read	Active tags can be expensive Requires external infrastructure

**Table 5:** Commercially available intelligent packaging and active packaging systems, reproduced with permission from [98].

Smart Packaging System	Sensors Type	Trade Name (Manufacturer)
Intelligent packaging systems	Integrity indicator (leak)	O_2_ Sense ^TM^ (Freshpoint Lab) Novas^®^ (Insignia Technologies Ltd.) Ageless Eye^®^) (Mitsubishi Gas Chemical Inc.) Timestrip^®^ (Timestrip Ltd.) Novas^®^ (Insignia Technologies Ltd.)
	Freshness	Freshtag^®^ (COX Technologies) Sensorq^®^ (DSM NVAnd Food Quality Sensor International)
	Temperature	Timestrip^®^ PLUS Duo (Timestrip UK Ltd.)
	Time-temperature	Timestrip Complete^®^ (Timestrip UK Ltd.) Monitormark^tm^ (3M^TM^, Minnesota) Fresh-Check^®^ (Temptime Corp) Onvu^tm^ (Ciba Specialty Chemicals and Freshpoint) Checkpoint^®^ (Vitsab) Cook-Chex (Pymah Corp) Colour-Therm (Colour Therm) Thermax (Thermographic Measurements Ltd.)
	RFID	Easy2log^®^ (CAEN RFID Srl) Intelligent Box Mondi Plc CS8304 (Convergence Systems Ltd.) Temptrip (Temptrip LLC)
Active packaging systems	Oxygen scavenger	Ageless (Mitsubishi Gas Chemical Co. Ltd., Japan) Freshilizer (Toppan Printing Co. Ltd., Japan) Freshmax, Freshpax, Fresh Pack (Multisorb Technologies, USA) Oxyguard (Toyo Seikan Kaisha Ltd., Japan) Zero_2_ (Food Science Australia, Australia) Bioka (Bioka Ltd., Finland)
	Moisture absorber	Dri-Loc^®^ (Sealed Air Corporation, USA) Tenderpac^®^ (SEALPAC, Germany)
	Antimicrobial packaging	Biomaster^®^ (Addmaster Limited, USA) Agion^®^ (Life Materials Technology Limited, USA)
	Ethylene scavenger	Neupalon (Sekisui Jushi Ltd., Japan) Peakfresh (Peakfresh Products Ltd., Australia) Evert-Fresh (Evert-Fresh Corporation, USA)

## Data Availability

Not applicable.
